# Patient-Facing AI-Enabled Digital Health Technologies and Quality of Life in Cancer: Systematic Review and Exploratory Meta-Analysis

**DOI:** 10.2196/94793

**Published:** 2026-08-10

**Authors:** Anica Ilic, Lene Kristine Juvet, Patrick Cairns, Kristen Elizabeth Thompson Thornton, Hanne Cathrine Lie

**Affiliations:** 1 Department of Behavioural Medicine, Institute of Basic Medical Sciences, Faculty of Medicine, University of Oslo, Postboks 1111 Blindern, Oslo, 0317, Norway; 2 Division of Infection Control, Norwegian Institute of Public Health, Oslo, Norway; 3 Department of Nursing and Health Sciences, Faculty of Health and Social Sciences, University of South-Eastern Norway, Borre, Norway

**Keywords:** neoplasms, AI, digital health, quality of life, patient-reported outcome measures

## Abstract

**Background:**

Cancer affects multiple physical, psychological, and social aspects of an individual’s life. Cancer survivors frequently report unmet needs long after diagnosis and require ongoing support. AI is increasingly embedded in patient-facing digital health technologies (DHTs) in oncology, yet its impact on different domains of patients’ and survivors’ health-related quality of life (HRQOL) remains unclear.

**Objective:**

This systematic review aims to (1) examine how AI has been integrated into patient-facing DHTs designed to support cancer survivors, (2) narratively synthesize the potential effects of these technologies on HRQOL and provide preliminary quantitative estimates through an exploratory meta-analysis, and (3) explore broader changes in additional patient-reported outcomes (PROs; secondary aim).

**Methods:**

PubMed, PsycINFO, Embase, Scopus, CINAHL, and the Cochrane Library were searched for articles published between January 2020 and August 2025. Reference lists of included articles were hand-searched for additional eligible studies. Eligible studies enrolled cancer survivors of any age and disease stage, evaluated a patient-facing DHT with AI components, and assessed HRQOL. Nonoriginal research and non-English reports were excluded. Risk of bias was assessed in all controlled studies using RoB 2 (revised Cochrane risk of bias 2) or ROBINS-I V2 (Risk of Bias in Non-Randomized Studies—of Interventions, Version 2), according to study design. Data on HRQOL and other PROs were synthesized narratively, and exploratory random-effects meta-analyses were conducted for HRQOL domains.

**Results:**

Eight reports from 7 studies from China and the United States (N=2867 participants) met the inclusion criteria, and 3 (n=292 participants) contributed to the exploratory meta-analysis. All studies included adults with various cancers at different stages and times since diagnosis. Most studies showed low risk of bias or some concerns (RoB 2), but one was evaluated as having a serious risk of bias (ROBINS-I V2). AI applications ranged from symptom monitoring to targeted education. The narrative synthesis suggested positive effects on selected HRQOL domains, particularly general health, with more pronounced effects in studies conducted in China. Exploratory meta-analyses demonstrated provisional moderate positive effects on global health (Hedges *g*=0.77, 95% CI 0.15‐1.40) and social functioning (Hedges *g*=0.75, 95% CI 0.08‐1.42), but no effects on physical functioning, role functioning, or emotional well-being. Other PROs indicated generally high user satisfaction and adherence, improved mental health outcomes, and reductions in physical symptoms. Only minor and mild adverse events were reported.

**Conclusions:**

Current evidence, although limited, suggests that AI-enabled patient-facing DHTs may benefit survivors’ HRQOL and other PROs, particularly in early survivorship. However, our findings are based on small and heterogeneous studies and should therefore be interpreted with caution. Robust trials with adequate sample sizes, longer follow-up, and appropriate control conditions, including DHTs without AI components, are needed to determine the specific contribution of AI.

## Introduction

Cancer affects not only physical health but also the emotional, social, and psychological well-being of patients and their close networks across the cancer trajectory [1]. Cancer survivors often face symptoms, lifestyle adjustments, and report information needs that persist long after cancer treatment [2]. Although cancer survivors value continued personal follow-up care [2,3], it is rarely achievable in routine practice due to limited health care resources, competing priorities, geographic barriers, and the growing number of individuals living with and beyond cancer [4]. These challenges highlight the need for scalable solutions that can complement traditional survivorship care.

Patient-facing digital health technologies (DHTs), such as mobile apps and digital therapeutics, may help bridge these gaps by delivering accessible, personalized, and timely support to patients in diverse settings [5]. Unlike DHTs designed for health care professionals, patient-facing DHTs are directly operated by patients themselves, enabling self-monitoring, symptom reporting, psychoeducation, and behavioral interventions in daily life [5]. Incorporating AI components into DHTs might further enhance their potential impact [6,7]. For instance, machine learning (ML) and natural language processing (NLP) can facilitate the processing and interpretation of large volumes of heterogeneous data, including symptom reports, sensor data, and medical records [8]. These technologies can thereby enable patient-facing DHTs to provide tailored feedback, adapt digital interventions to individual needs, or identify moments when users are most receptive to receiving an intervention [8,9]. However, these potential benefits should be weighed against important concerns. Embedding AI into patient-facing DHTs may introduce additional risks to data privacy and exacerbate existing gaps in digital access [10], and consequently in access to health care. Furthermore, understanding how patients perceive and use these tools is necessary, as their experience will strongly influence engagement and uptake [11].

Although interest in patient-facing DHTs is increasing, few trials examine their effects from the patients’ perspective [12]. Patient-reported outcomes (PROs) are critical indicators of how individuals perceive their health, functioning, and quality of life, as well as the impact of interventions on their daily lives [13]. PROs capture dimensions of health that cannot be observed or measured by clinicians alone and are particularly relevant in chronic and oncological conditions where maintaining quality of life is a primary treatment goal [14]. Health-related quality of life (HRQOL) represents a key multidimensional construct spanning physical, psychological, and social domains [15,16]. Various instruments exist to assess HRQOL, ranging from generic measures to cancer-specific scales, each assessing different facets of survivors’ lived experiences [15,17]. However, it remains unclear whether AI-enabled DHTs adequately address the complex physical, emotional, and social needs of cancer survivors [18]. To address this gap, this systematic review aims to (1) examine how AI has been integrated into patient-facing DHTs designed to support cancer survivors, (2) narratively synthesize the potential effects of these technologies on HRQOL and provide preliminary quantitative estimates through an exploratory meta-analysis, and (3) explore broader changes in additional PROs (secondary aim).

## Methods

This systematic review followed the PRISMA (Preferred Reporting Items for Systematic Reviews and Meta-Analyses) 2020 guidelines (Checklist 1) [19]. The protocol, including a detailed description of the search strategy, is registered in the PROSPERO systematic review registry (CRD420251021466).

### Database Search

The search strategy consisted of 4 concept blocks (cancer, AI, DHT, and HRQOL) and combined free-text and thesaurus terms using AND/OR Boolean operators. Initial terms were identified by the first author (AI) through scoping searches and key articles in the field. The strategy was refined iteratively in consultation with a co-author (HCL) to capture relevant synonyms, acronyms, and spelling variants, and it was reviewed by an experienced librarian. Search strings were adapted for each database, and we focused on articles containing any of the terms in the title, abstract, and, where possible, the keywords. No further filters or language restrictions were applied at the search stage.

We conducted an initial search on May 20, 2025, in PubMed, PsycINFO, Embase, Scopus, CINAHL, and the Cochrane Library, covering records published from January 1, 2020, to the search date. The start date (January 1, 2020) was chosen to account for the rapid development of AI and to ensure the inclusion of the most recent evidence [20,21]. A second search was conducted in the same databases on August 26, 2025, adding 3 terms that had not been included previously. Therefore, we obtained a large number of duplicates. The reference lists of all included publications were screened to identify additional potentially relevant studies.

### Eligibility Criteria

Reports were eligible if they (1) enrolled cancer survivors at any disease stage, including those undergoing active treatment or palliative care (in accordance with the definition of the National Cancer Institute) [22], (2) evaluated patient-facing DHTs that explicitly included AI components (AI-enabled DHTs), and (3) reported an assessment of HRQOL. For the purposes of this review, AI-enabled DHTs refer to patient-facing DHTs that incorporate an AI component, whether embedded within the platform or operating as an external module.

Nonoriginal reports, including other reviews, editorials, commentaries, conference abstracts, and study protocols, were excluded. Only studies that included a control group (eg, usual care and non–AI-enabled DHTs) were eligible for inclusion in the meta-analysis.

Although we initially considered including non-English articles relying on software-generated translations, these were ultimately excluded because we could not validate them with a native speaker and therefore could not avoid potential misinterpretation. However, these studies are cited in the results for completeness.

### Study Selection

All identified references were screened using Covidence (Veritas Health Innovation Ltd) [23], and duplicates were automatically excluded. Two authors (AI and PC or KETT) independently screened all titles and abstracts against previously agreed-on inclusion and exclusion criteria. The same 2 authors screened the full texts of all articles previously included by at least one author to make a final decision on inclusion. Any disagreements were resolved through discussion or by consulting a third author. The study selection process is summarized in a PRISMA 2020 flow diagram.

### Data Extraction

Data extraction was supported using SciSpace [24] and Perplexity [25]. All machine-extracted information was verified against the original manuscripts to ensure that no relevant information was misinterpreted or missed (AI) and validated by a second author (PC or KETT). Data were extracted from the published articles and any supplementary materials, and no individual participant data were requested. Where information was unclear or insufficiently detailed, we contacted the authors for clarification. However, no responses were received.

Collected data included (1) study information: title, authors, year, journal, country, and study design; (2) sample characteristics: sample size, sociodemographic information, and cancer-related characteristics for both intervention and control groups; (3) DHT details: type, purpose, AI components, input data sources, and output type; (4) HRQOL outcomes: baseline and postintervention measurements, instruments used, domains assessed, and main findings; and (5) additional PROs: instruments used and key results. When multiple articles reported on the same study, all relevant publications were considered to provide a comprehensive description of the methods and outcomes.

### Risk of Bias

For randomized controlled trials (RCTs), the risk of bias was assessed using the revised Cochrane Risk of Bias 2 (RoB 2) tool [26], and for non-RCTs, the ROBINS-I V2 (Risk of Bias in Non-Randomized Studies—of Interventions, Version 2) [27]. Single-arm studies were not formally assessed for risk of bias and were reported narratively without inclusion in any quantitative synthesis.

Two authors (AI and LKJ) independently evaluated each study across all domains, with possible ratings of low risk, some concerns, or high risk (RoB 2), or low, moderate, serious, and critical concerns (ROBINS-I V2). The evaluations were then compared, and any discrepancies were resolved through discussion. When multiple articles reported on the same study, all relevant publications were considered to provide a comprehensive assessment.

### Data Synthesis

For the purposes of this systematic review, studies were grouped as controlled (including randomized and nonrandomized trials) or single-arm studies. Controlled studies were eligible for quantitative synthesis (meta-analysis), whereas single-arm studies were only summarized narratively.

#### Narrative Synthesis

HRQOL outcomes were summarized using a narrative synthesis approach (AI) [28]. Given the small number of studies included, thematic clustering was not feasible. Instead, we presented results on a study-by-study basis, describing the reported direction and highlighting any patterns across interventions where possible. Similarly, we used a descriptive narrative approach to describe PROs beyond HRQOL.

#### Meta-Analysis

Because HRQOL instruments varied across studies, we extracted all domains reported at all time points and compared the underlying items to align conceptually similar domains across different instruments (Multimedia Appendix 1). We pooled outcomes only for domains that were conceptually comparable across instruments. For all instruments, higher scores indicated higher HRQOL. When studies reported multiple follow-up time points, we included the assessment closest to the end of the intervention to minimize heterogeneity in exposure duration.

For controlled studies, we conducted exploratory meta-analyses using random-effects models due to the clinical and methodological heterogeneity across digital health interventions and populations with cancer. For continuous HRQOL outcomes, provisional effect sizes were calculated as standardized mean differences (Hedges *g*) with 95% CIs. When authors reported SEs, we converted them to SDs to ensure comparability across studies. When the reported variability measure was not specified as SD or SE, it was assumed to represent SD, as SD is more commonly reported in HRQOL research. When studies reported both intention-to-treat and per-protocol analyses, we used data from the intention-to-treat analyses.

Statistical heterogeneity was assessed using the *I*^²^ statistic. Sensitivity analysis was performed using a leave-one-out approach. Results of individual studies and pooled effect estimates were visually displayed using forest plots. Study characteristics and extracted outcome data were summarized using tables. All analyses were performed using the *meta* command suite in Stata (version 9.5; StataCorp LLC) [29].

## Results

### Study Selection

**Figure 1.**
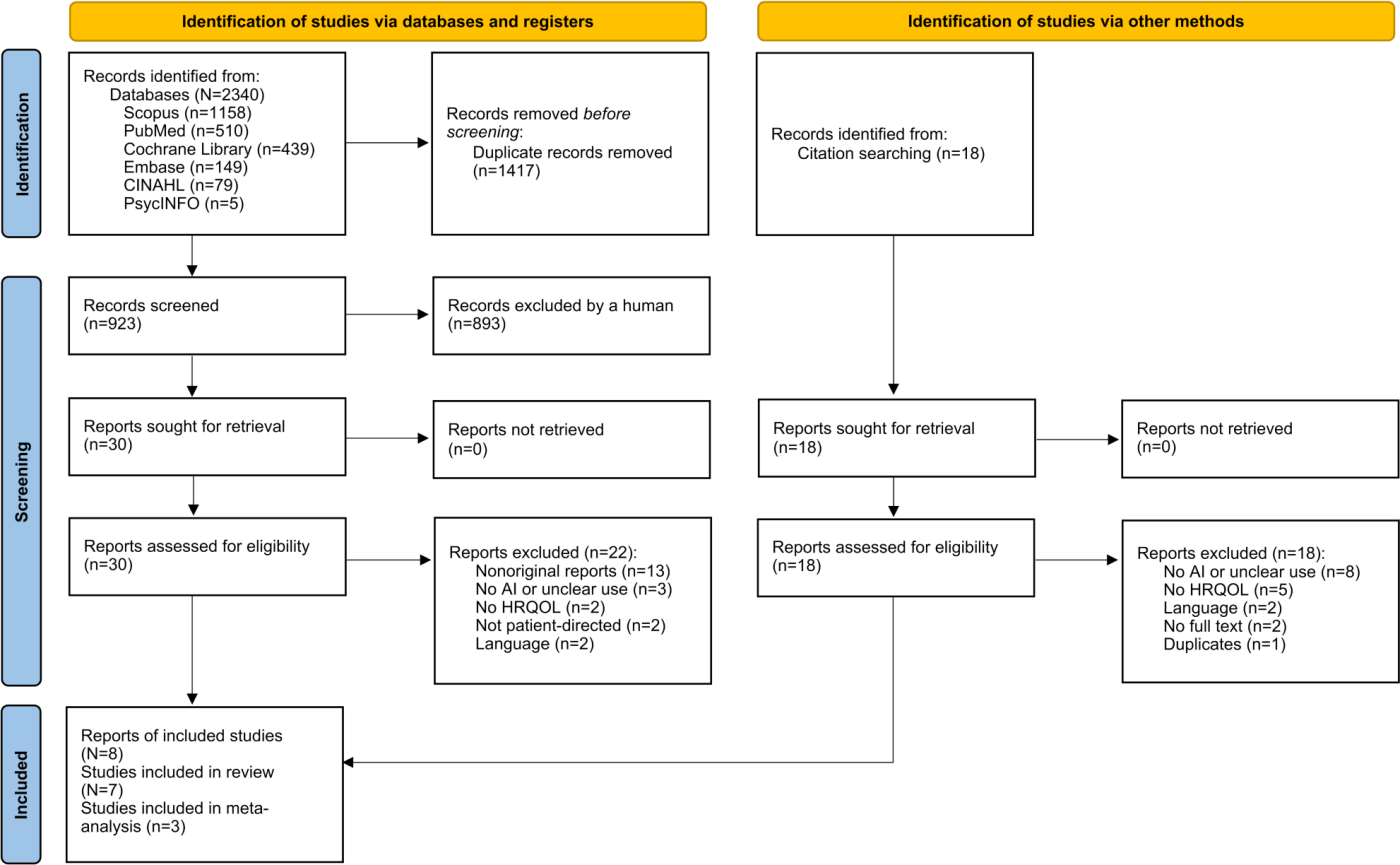
PRISMA (Preferred Reporting Items for Systematic Reviews and Meta-Analyses) 2020 flow diagram. HRQOL: health-related quality of life. Source: [19].

A comprehensive search of 6 scientific databases identified 2340 records (Figure 1). After removing duplicates (n=1417), 923 records were screened. Of these, 30 full-text articles were assessed for eligibility, and 8 publications from 7 studies (N=2867 participants) met the inclusion criteria and were included in the review.

Two potentially relevant records published in Chinese were excluded because reliable translation and validation were not feasible. One was a pilot study from an already included study [30], and the other reported data from a distinct study not otherwise included in this review [31]. Citation searching did not identify any additional eligible studies.

### Study Characteristics

The 7 included studies comprised 2 single-arm studies [32,33], 3 RCTs [34-37], 1 partial-crossover RCT [38], and 1 non-RCT [39] (Table 1).

The studies covered multiple cancer types and survivorship stages (Table 1). RCTs by Schmitz et al [38] in the United States and by Hu et al [34] and Jiang et al [35] in China focused exclusively on breast cancer survivors, while a US single-arm study by Horesh et al [33] included both breast and ovarian cancer survivors. Together, these studies targeted survivorship stages from active treatment through early survivorship to metastatic disease. The RCT by Li et al [37] examined early-stage postoperative lung cancer survivors in China, and the RCT by Kamdar et al [36] focused on patients with metastatic solid tumors receiving palliative care in the United States. The remaining studies included survivors of any cancer type in different contexts. The non-RCT by Zhang et al [39] focused on patients receiving chemotherapy in China, and the single-arm study by Buchan et al [32] included individuals at any stage of disease in the United States. Quality of life was assessed in all included studies as a primary or secondary outcome, and most studies used validated HRQOL instruments.

**Table 1.** Study characteristics^a^.

Study	Study characteristics	Assessment details	Sample^b^	Cancer details	HRQOL^c^ measure
Buchan et al [32], 2024	United States; single-arm cohort study	Within-group comparison; monthly assessments (T0, 49.1 mo; median follow-up 8.8 mo)	2423	Mixed cancers; any stage	Not specified
Horesh et al [33], 2022	United States; single-arm pre-post study	Within-group comparison; 2 assessments (T0, 24 d)	37	Breast and ovarian cancers; on active treatment, experiencing hot flashes	WHOQOL-BREF^d^
Hu et al [34], 2025; Jiang et al [35], 2024^j^	China; RCT^e^	General information online; 3 assessments (T0, 1 mo, 3 mo)	115 (intervention=60 and control=55)	Breast cancer; early survivorship	FACT-B^f^
Kamdar et al [36], 2024	United States; RCT	Usual care; 3 assessments (T0, 4 wk, 8 wk)	79 (intervention=39 and control=40)	Mixed (solid) cancers; metastatic, receiving palliative care	FACT-G^g^
Li et al [37], 2025	China; RCT	Usual care; 2 assessments (T0, 5 mo)	36 (intervention=20 and control=16)	Lung cancer (NSCLC)^h^; early survivorship, postoperative	EORTC QLQ-C30^i^
Schmitz et al [38], 2023^j^	United States; RCT, partial crossover	Delayed intervention; 3 assessments (T0, 3 mo, 6 mo^k^)	33 (intervention=17 and control=16)	Breast cancer; metastatic	SF-36^l^
Zhang et al [39], 2025^j^	China, non-RCT	Non-RCT; usual care; 2 assessments (T0, 6 mo)	144 (intervention=72 and control=72)	Mixed cancers; receiving chemotherapy	EORTC QLQ-C30

a In total, 8 publications from 7 studies were included in this review.

b Sample sizes reported in this table reflect the number of participants included in the HRQOL analyses (including intervention and control groups). They may differ from the sample sizes reported elsewhere in the publications.

c HRQOL: health-related quality of life.

d WHOQOL-BREF: World Health Organization Quality of Life Scale–Brief version.

e RCT: randomized controlled trial.

f FACT-B: Functional Assessment of Cancer Therapy–Breast.

g FACT-G: Functional Assessment of Cancer Therapy–General.

h NSCLC: non–small cell lung cancer.

i EORTC QLQ-C30: European Organisation for Research and Treatment of Cancer Quality of Life Questionnaire–Core 30.

j Included in the meta-analysis.

k Immediate group: intervention 0‐6 months; delayed group: 3‐6 months; control assessed 0‐3 months prior to intervention.

l SF-36: 36-item Short-Form Health Survey.

### Risk of Bias

**Figure 2.**
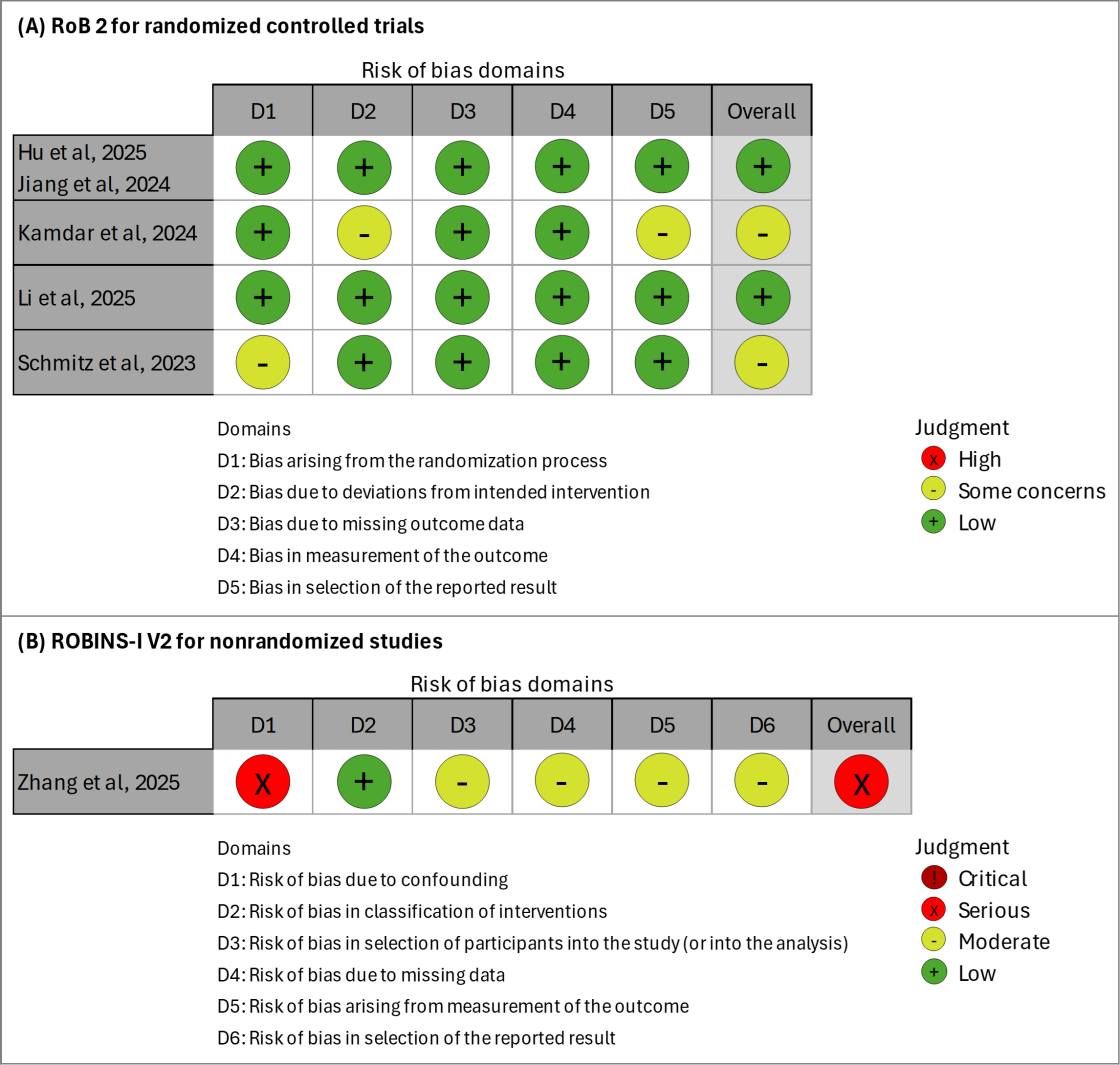
Risk of bias assessment: (A) RoB 2 (Revised Cochrane risk of bias 2) for randomized controlled trials, (B) ROBINS-I V2 (Risk of Bias in Non-Randomized Studies—of Interventions, Version 2) for nonrandomized studies [34-39].

Of the 4 RCTs, 2 were rated as low risk of bias, and 2 studies were assessed as having some concerns. However, all concerns were observed in different domains and did not indicate a consistent source of bias across the evidence base. The non-RCT by Zhang et al [39] was found to be at serious risk of bias, primarily due to inadequate control of confounding factors in a time-separated convenience sample. There were additional concerns regarding the unclear management of exclusions and missing data. Furthermore, inconsistencies in the reporting of results suggest caution when interpreting the findings. No studies were evaluated as high (RoB 2) or critical (ROBINS-I V2) risk of bias. Therefore, none were excluded. Further details on the risk of bias evaluation are available in Figure 2.

### Aim 1: AI-Enabled DHTs for Patient Support in Cancer

#### Purpose

Most of the DHTs included in this review aimed to provide home-based support for monitoring and managing physical symptoms associated with cancer treatment, such as pain, nausea, fatigue, and sleep disturbances [33-36,38,39]. Three studies addressed psychosocial needs by offering supportive care [33-35,38]. Two studies specifically targeted cardio-oncology rehabilitation [37] or nutritional support for cancer survivors [32] (Table 2).

**Table 2.** Description of digital health technologies.

Study	Name	Purpose
Buchan et al [32], 2024	Ina	AI-driven virtual text message–based nutrition assistant providing evidence-based, personalized nutritional counseling, recipes, and symptom management support to improve dietary habits, symptom control, and HRQOL^a^ for diverse cancer types.
Horesh et al [33], 2022	Bubble	AI-VR^b^ intervention, delivered via mobile apps (Bubble and Luna) and VR goggles, with elements of cognitive behavioral therapy and mindfulness-based stress reduction, to reduce hot flash frequency and severity, alleviate psychological distress, improve sleep quality, and enhance HRQOL in women with breast and ovarian cancers. The intervention takes place in the “Frosty” virtual environment and is led by an avatar named Luna.
Hu et al [34], 2025; Jiang et al [35], 2024	AI-TA	AI-driven intelligent interactive mobile app designed to reduce physical, psychological, and social premature frailty, thereby improving HRQOL among young breast cancer survivors through personalized content delivery, symptom tracking, and 2-way web-based follow-up with health care professionals.
Kamdar et al [36], 2024	ePAL	Digital therapeutic mobile app designed to manage cancer-related pain in patients with advanced cancer through active pain monitoring, an AI algorithm that triages patient symptoms and identifies specific barriers to pain control, patient education modules, and real-time clinician outreach to address identified barriers to pain management.
Li et al [37], 2025	Recovery Plus Health (Recovery Plus USA Inc)	Cardio-oncology rehabilitation program delivered via the Recovery Plus mobile app using AI-driven exercise prescriptions with real-time heart rate monitoring to improve cardiopulmonary fitness, reduce cancer-related symptoms (fatigue, anxiety, and daytime dysfunction), and enhance HRQOL in early-stage lung cancer survivors.
Schmitz et al [38], 2023	Nurse AMIE	Conversational AI–based supportive care intervention delivered via Amazon Echo Show with Alexa to address symptoms (pain, fatigue, sleep disturbance, and distress), provide nutritional guidance, and improve HRQOL in patients with metastatic breast cancer.
Zhang et al [39], 2025	Not specified	Deep learning platform for symptom management designed to provide continuous, personalized symptom management for patients with cancer undergoing chemotherapy, aiming to reduce anxiety and depression and improve HRQOL by delivering real-time nursing support, symptom monitoring, and medical guidance outside hospital visits.

a HRQOL: health-related quality of life.

b VR: virtual reality.

#### Delivery Modality

Of the 4 interventions delivered via mobile applications, 2 were application-only [34-36], and 2 were delivered in combination with wearable devices such as virtual reality (VR) goggles [33] or heart rate bands [37]. The remaining interventions were delivered through web platforms or portals accessible on both computers and smartphones [39], via text messaging [32], or through a voice-based virtual assistant [38].

Six interventions included a human component alongside the DHTs. Three interventions encouraged participants who reported high symptoms to contact the health care team [32,37,38], and 2 automatically notified the health care team when any alarming symptoms were observed [36,38]. In 2 interventions, health care professionals supervised AI-driven recommendations and adjusted them when necessary [32,37]. Two DHTs also encouraged questions and peer group discussions [34,35,39].

#### AI Components

Across the interventions, AI was mainly used to personalize and optimize supportive care delivery. NLP was used for text analysis of patient input and for generating personalized responses, enabling the development of conversational interfaces [32,35,37]. ML and predictive analytics were used to predict patient needs, triage symptoms, and adapt interventions in real time [32,35,36,39]. Knowledge graphs and expert systems provided structured decision support, ensuring evidence-based recommendations [32,35,36]. Recommender systems combined keyword analysis and filtering to personalize the delivered content [33-35]. Other DHTs included AI-driven exercise prescription systems that adjusted training parameters based on wearable data [37], as well as speech recognition for voice-interactive virtual assistants [38].

#### Input Sources and Output Types

The included studies can be classified into 3 groups. First, several interventions in breast cancer used patient-reported symptoms, survey data, and basic clinical history to provide personalized education, symptom-management guidance, and supportive follow-up [33-35,38]. Second, studies focused on more intensive symptom management in people with advanced cancer or those receiving palliative care used repeated symptom reporting, clinical records, and real-time monitoring to trigger tailored coaching, clinician outreach, or escalation [36]. Third, studies centered on nutrition or broader supportive care used baseline medical and nutrition history, treatment details, and user input to deliver individualized advice, recipes, nursing guidance, and interactive messaging [32,37,39].

### Aim 2: Effect on HRQOL

Multimedia Appendix 2 presents the magnitude and direction of changes for all presented outcomes.

#### Narrative Synthesis of HRQOL Findings

Across 5 controlled studies, the 3 studies conducted in China reported improvements in at least one HRQOL domain, while the 2 US studies found no significant changes. Li et al [37] found that the intervention group showed improved global health status compared with usual care at 5 months postintervention, although no between-group differences were observed across subdomains. Zhang et al [39] reported significant improvements across nearly all HRQOL domains in the intervention group, including overall health status, role functioning, cognitive functioning, emotional functioning, and social functioning. Physical functioning (sometimes referred to by the authors as “somatic function”) was the only domain that did not differ significantly between groups at 6 months postintervention [39]. Jiang et al [35] reported that although some HRQOL domains did not change significantly at 1-month postdiagnosis, all domains, including overall HRQOL, physical, social or family, emotional, and functional well-being, as well as breast cancer-specific concerns, showed significant between-group improvements by 3 months. Hu et al [34], reporting on the same study population, similarly found that total HRQOL improved significantly more in the intervention group than in the control group over this period. In the RCTs conducted by Kamdar et al [36] and Schmitz et al [38] in the United States, no significant effects of the intervention on HRQOL were observed.

The 2 single-arm studies conducted in the United States showed similar results. Buchan et al [32] reported that 81% of users experienced some improvement in their HRQOL after using the Ina virtual dietitian. Horesh et al [33] observed within-group improvements in physical and psychological domains 24 days after the intervention, with no changes in social or environmental domains.

#### Quantitative Synthesis of HRQOL Findings

From the 7 identified studies, 3 (n=292) contributed to the exploratory meta-analysis examining the effect of AI-enabled DHTs on HRQOL [35,38,39]. Studies were excluded from the meta-analysis for the following reasons: single-arm studies (n=2) [32,33], lack of quantitative HRQOL data (n=1) [36], reporting HRQOL change scores without corresponding pre- and postintervention values (n=1) [37], and multiple publications from the same study (n=1) [34]. For studies with incomplete HRQOL data, we contacted the authors but received no response. In cases of duplicate publications, the report providing the most comprehensive HRQOL data was used [35]. The 3 studies included in the meta-analysis were judged to have low [34,35], some concerns [38], and serious risk of bias [39], primarily due to potential confounding.

**Figure 3.**
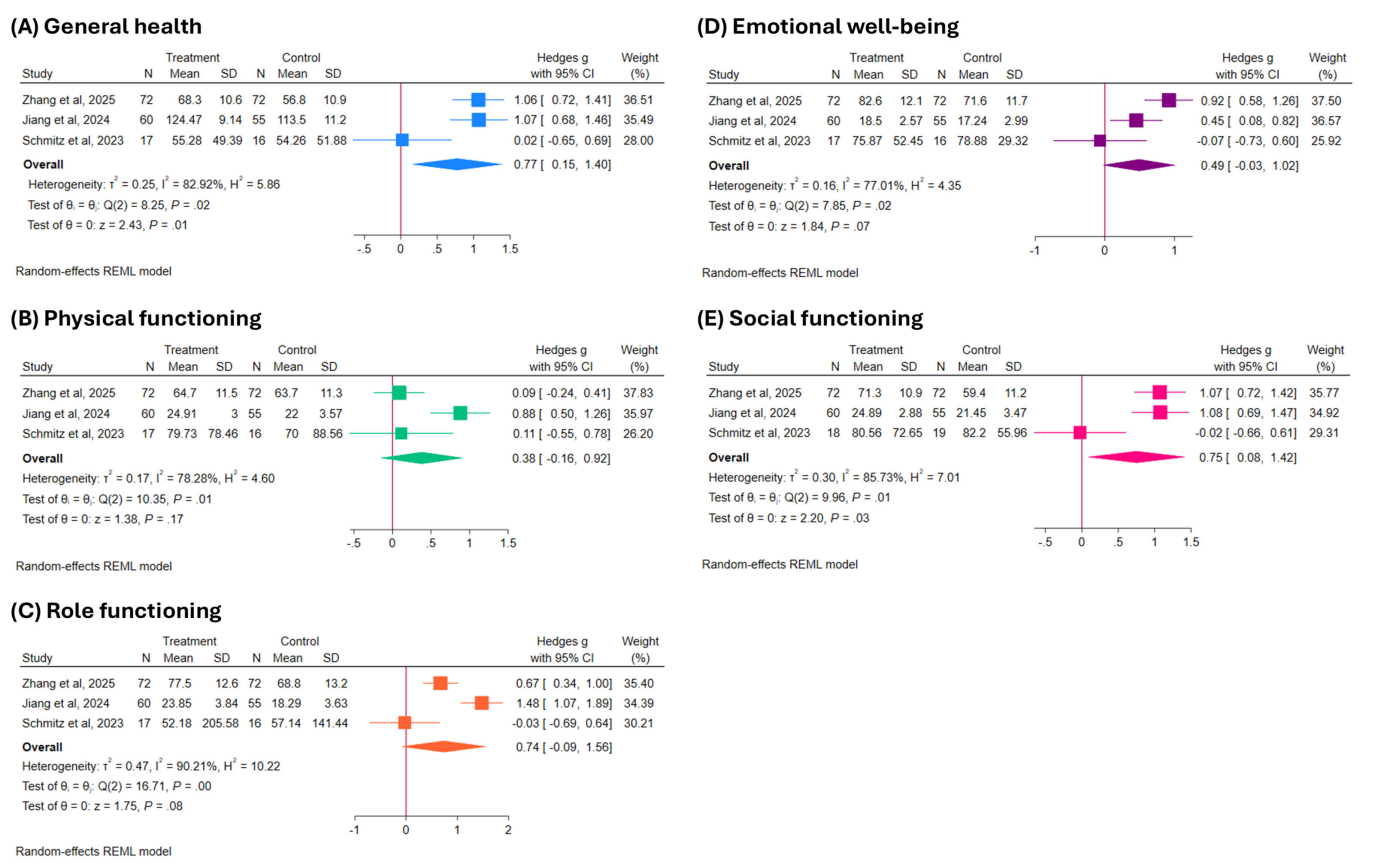
Forest plots of pooled mean differences in various health-related quality of life domains: (A) general health, (B) physical functioning, (C) role functioning, (D) emotional well-being, and (E) social functioning [35,38,39]. REML: restricted maximum likelihood.

Five HRQOL domains were analyzed: general health, physical functioning, role functioning, emotional well-being, and social functioning. In the exploratory meta-analyses, statistically significant pooled effects were found for general health (Hedges *g*=0.77, 95% CI 0.15 to 1.40; *P*=.01; *I*^²^=82.9%) and social functioning (Hedges *g*=0.75, 95% CI 0.08 to 1.42; *P*=.03; *I*^²^=85.7%), both indicating moderate effect sizes. However, the high heterogeneity across these analyses warrants cautious interpretation. No statistically significant effects were found for physical functioning, role functioning, or emotional well-being (see Figure 3 for estimates).

Leave-one-out sensitivity analyses indicated that the pooled effect was sensitive to individual studies for 4 of the 5 HRQOL domains. Specifically, excluding the US study by Schmitz et al [38] increased effect sizes for general health, role functioning, emotional well-being, and social functioning, while physical functioning remained stable across all exclusion scenarios (full results are provided in Multimedia Appendix 3). Excluding the studies by Zhang et al [39] or Jiang et al [35] did not change the statistical significance of the pooled effects for any domain.

Due to the small number of included studies, other subgroup analyses, as well as formal tests for publication bias (Egger regression) and visual inspection of funnel plots, were not feasible [26].

### Aim 3: Changes in Other PROs

In addition to HRQOL, the included studies reported other PROs, including user satisfaction and adherence, mental health, physical symptoms, additional PROs, and adverse effects.

#### User Satisfaction and Adherence

Five studies reported outcomes related to satisfaction or acceptability [32,33,36,38,39]. Across these studies, users consistently rated the DHTs positively, with high scores for ease of use, clarity, and overall experience. Satisfaction levels generally exceeded 70%, and usability assessments indicated strong acceptability of the DHTs. Most users found the content helpful and the DHTs easy to navigate. However, in Zhang et al [39], a small proportion reported difficulties with navigation, internet access, or understanding specific content.

Adherence and engagement with the DHTs varied across studies. Overall, most users engaged with the DHTs at least occasionally, though the frequency and consistency of engagement differed by intervention type and activity. High adherence rates were reported in 2 interventions targeting early survivorship in China, with 88% to 97% of participants completing prescribed sessions or following recommended activities [34,37]. Engagement with DHTs targeting patients with advanced cancer in the United States declined gradually over time, with weekly use dropping from initially high rates to lower levels by later weeks [36,38]. Furthermore, specific features within single DHTs were used at varying rates. For example, most participants used nutritional tips, whereas fewer used the suggested recipes in the virtual dietitian intervention by Buchan et al [32].

#### Mental Health

Across the included studies, 4 of 6 interventions that reported mental health outcomes showed improvements. The US single-arm hot flash intervention study reported significant reductions in perceived stress, general psychological distress, and illness perceptions from pre- to postintervention [33]. Another study targeting women with breast cancer in China showed greater decreases in psychological frailty, psychological distress, symptom frequency, and global distress in the intervention group compared with controls on follow-up [34,35]. However, the US study by Schmitz et al [38] showed no significant between-group differences in distress among metastatic breast cancer survivors. Additional evidence indicated mixed effects for anxiety. Two controlled studies from China found decreases in anxiety. While Zhang et al [39] reported greater improvement in the control group than in the intervention group, Li et al [37] found the opposite. In addition, the RCT in US patients with metastatic cancer by Kamdar et al [36] found that anxiety worsened in the intervention group compared with the control group. Depression was assessed only in the study by Zhang et al [39] involving patients with mixed cancer undergoing chemotherapy in China, which found greater improvements in the intervention group than in the control group.

#### Physical Symptoms

Across the included studies, several DHTs showed improvements in physical symptoms. The virtual dietitian intervention by Buchan et al [32] reported high perceived usefulness for symptom management, with approximately one-third of users showing reductions in both symptom count and total symptom severity over time. Significant reductions in hot flash interference and frequency, along with improvements in overall sleep quality, sleep onset latency, and subjective sleep quality were observed in a US hot flash intervention [33]. Similarly, a controlled study to manage premature frailty in young breast cancer survivors from China showed greater reductions in fatigue and physical symptoms, and an increase in physical activity in the intervention group, although group-by-time effects were not significant [34]. The RCT by Kamdar et al [36] found significant reductions in pain severity over 8 weeks, with a higher proportion of participants achieving substantial pain improvement compared with the control group. Additional benefits were reported in fatigue and daytime dysfunction, following the digital cardio-oncology rehabilitation program by Li et al [37]. One controlled study of US metastatic breast cancer survivors found no significant between-group differences in pain, sleep, and fatigue [38].

#### Other PROs

Other PROs were examined in the studies by Hu et al [34] and Jiang et al [35], which evaluated social support and self-efficacy following a digital humanistic program to manage premature frailty in young breast cancer survivors. Social support increased over time in both the intervention and control groups, with greater improvement observed in the intervention group [34,35]. In addition, Jiang et al [35] reported an increase in self-efficacy among participants receiving the intervention.

#### Adverse Effects

Across the included studies, adverse events were occasionally reported and generally mild. A small proportion of participants experienced cybersickness in the AI-VR intervention by Horesh et al [33] for the management of hot flashes. In the premature frailty trial conducted in China, adverse changes were uncommon, with only a few participants showing increases in symptom levels or declines in HRQOL [34]. A US intervention for pain management in patients with advanced cancer showed a small but statistically significant increase in anxiety symptoms in the intervention group, although values remained within the mild range and below clinically significant thresholds [36]. No adverse effects were identified in 2 studies [37,38], and the remaining manuscripts did not report adverse events [32,35,39].

## Discussion

### Principal Findings

This systematic review synthesized evidence on available patient-facing AI-enabled DHTs and their effect on HRQOL and broader PROs in cancer populations. Eight publications from 7 studies conducted in China and the United States evaluated a range of mobile- and web-based interventions, most of which aimed to support at-home symptom management. AI was used to personalize recommendations (eg, exercise, nutrition, and symptom triage), adapt delivery formats (eg, VR and voice interfaces), and tailor educational content. Overall, the effects on HRQOL were mixed. Results from the narrative synthesis showed that among the 5 controlled studies, all 3 RCTs conducted in China found significant improvements across multiple HRQOL domains, including global health status, role, cognitive, emotional, social, and functional well-being, while neither of the 2 US RCTs found significant between-group effects. The 2 single-arm US studies suggested within-group improvements, although the absence of control groups limits the interpretability of those findings. An exploratory meta-analysis of 5 HRQOL domains showed modest improvements only in general health and social functioning, but no effect on physical functioning, role functioning, and emotional well-being. However, these results should be interpreted with caution given the small number of contributing studies, limited sample sizes, heterogeneity, and the presence of risk-of-bias concerns in individual studies. Other PROs, including mental health, physical symptoms, self-efficacy, and social support, also showed mixed but generally encouraging results. Mental health outcomes were variable, with some DHTs reducing distress or anxiety and others showing no effect or small, but not clinically significant, increases in anxiety. Despite considerable heterogeneity in both interventions and populations, most DHTs were associated with improvements in key physical symptoms, including pain, fatigue, and sleep. User adoption and satisfaction were generally high, and only mild adverse effects were reported occasionally.

Several patterns might help explain the variation in effects across studies and guide the design of future AI-enabled DHTs for cancer survivorship care. Improvements in HRQOL and other PROs were more frequent in the trials conducted in China, particularly for general health, whereas effects were less consistent across specific HRQOL domains. Two trials conducted in the United States showed no benefits in HRQOL, while 2 observational studies from the United States lacked control groups. Methodological factors, including small samples, heterogeneous measures, and short follow-up periods, likely contributed to these differences. In digital oncology research, changes in HRQOL might require sustained use of DHTs over time, as physical and psychological symptoms develop gradually [40,41]. Trials with short follow-up periods may, therefore, have lacked sufficient time to detect change, even when adherence was high. Another explanation might be differences in perceptions of AI-enabled DHTs and in-person consultations across cultures [42]. These may influence how patients interpret symptom feedback, interact with automated recommendations, and integrate digital tools into their routines. Furthermore, cross-national research suggests that social desirability bias varies across cultures, with relatively higher levels observed in China [43]. This may have led to more favorable evaluations of AI-enabled DHTs, especially in clinician-administered surveys. However, these cultural explanations remain speculative and cannot be distinguished from other country-level differences.

Our findings should be interpreted in the context of heterogeneous definitions of cancer survivorship across studies, which translate into substantial differences in symptom burden and care needs. While some interventions targeted patients with metastatic or advanced disease receiving ongoing or palliative treatment, others focused on disease-free survivors after curative therapy. Consistent with this heterogeneity, trials reporting no improvements in HRQOL tended to include patients with metastatic disease. Previous studies have shown that, among patients with high symptom burden in palliative care settings, supportive DHTs alone are often insufficient to improve overall HRQOL, although they may alleviate specific physical symptoms [44]. Our results have partially confirmed these findings. While AI-enabled tools may offer more personalized and proactive support than traditional DHTs, their benefits may be limited in advanced disease settings. This suggests that AI-enabled interventions should be integrated with comprehensive supportive care to improve HRQOL in advanced cancer.

Studies showing positive effects on HRQOL and PROs often included human involvement. This aligns with broader evidence that digital tools can initiate but rarely sustain long-term engagement among populations with complex needs without human involvement [45]. Hybrid models that combine AI-enabled monitoring and personalization with clinician or nurse follow-up leverage the strengths of both technology and human support. Thus, they can better translate digital engagement into behavior change and symptom improvement. Interventions that supported question-asking and peer discussion also reported positive effects on PROs. Features that enable communication with clinicians or connect patients with peers may increase self-efficacy, normalize experiences, and reduce isolation—mechanisms that are widely recognized in cancer survivorship research [46]. However, AI components themselves may introduce bias. For example, NLP or large language model–based systems that classify patient input or triage symptoms can introduce systematic measurement shifts, which could affect clinical decision support [47,48]. These findings highlight the value of designing comprehensive DHTs as platforms for holistic support.

User satisfaction and engagement were high across studies, indicating strong acceptability of DHTs containing AI components in cancer survivorship care. Notably, the effects observed on HRQOL and other PROs are similar to those reported for previous digital health interventions without AI [41,46]. However, because none of the studies included equivalent DHTs without AI components, we cannot determine whether these effects are attributable to the AI functionality, other DHT features, or nonspecific effects. Therefore, integrating AI might not significantly alter patients’ experiences, either by enhancing outcomes or introducing new barriers. Instead, the effectiveness of DHTs on PROs seems to depend primarily on design quality and relevance of support rather than on whether the underlying system relies on AI. Future research should focus on larger, multisite, longitudinal trials, more diverse populations with cancer, the use of traditional DHTs without AI components for control purposes, and the identification of different engagement trajectories among users. In practice, AI-enabled DHTs may support HRQOL when paired with adequate human follow-up, peer-support features, and careful patient selection based on disease stage, symptom burden, and digital readiness.

### Limitations

Our findings should be interpreted in light of several limitations. First, clinical heterogeneity limits the validity of pooled estimates. The included studies covered diverse types of cancer and stages of survivorship, making a single pooled estimate for cancer survivors problematic. Furthermore, the 3 different HRQOL instruments used across studies introduce measurement heterogeneity. Readers should, therefore, interpret findings from the exploratory meta-analyses with caution.

Second, the small number of eligible studies and participants limits the statistical power of the meta-analyses. While the narrative synthesis provides additional insights, the results from the meta-analyses should be considered exploratory.

Third, methodological limitations across studies reduce comparability and generalizability and may inflate observed effects. These limitations include small sample sizes, lack of control groups or randomization, short follow-up periods, and reporting limitations. None of the studies included equivalent DHTs without AI components, so it is not possible to determine whether the observed benefits are due to AI functionality, other DHT features, or nonspecific effects.

Fourth, geographic confounding limits generalizability. The limited geographical diversity (primarily studies from China vs US) prevents the isolation of intervention effects from country-level factors such as health care systems, cultural context, or implementation characteristics.

Fifth, how AI was defined and operationalized varied across studies, ranging from rule-based decision support to ML algorithms. This complicates direct comparisons across interventions and limits the interpretability of pooled findings. Furthermore, although the search strategy was intentionally broad and included multiple AI-related terms, studies that did not adequately describe the technical components of their DHTs may have been missed. Future reviews would benefit from applying a prespecified AI classification framework.

Finally, research on AI-enabled DHT is advancing rapidly. The numerous protocols identified during the screening process suggest that more publications are forthcoming. A living systematic review approach may, therefore, be valuable for maintaining an up-to-date evidence base.

### Conclusions

This review provides a timely synthesis of evidence on AI-enabled DHTs for cancer care and establishes a conceptual and methodological foundation for future work. Although effects on HRQOL were mixed, some interventions showed benefits across HRQOL domains and other PROs. Importantly, as none of the studies used control groups with equivalent DHTs without AI components, the observed benefits cannot be attributed specifically to the AI components. Our findings are preliminary, and it is too early to conclude whether patient-facing AI-enabled DHTs improve cancer survivorship care. Future research should employ active control conditions to isolate the contribution of AI features specifically.

## Supplementary material

10.2196/94793 Multimedia Appendix 1 Health-related quality of life domain comparison.

10.2196/94793 Multimedia Appendix 2 Changes in patient-reported outcomes.

10.2196/94793 Multimedia Appendix 3 Leave-one-out sensitivity analysis.

10.2196/94793 Checklist 1 PRISMA 2020 checklist.

## Abbreviations

DHT: digital health technology
HRQOL: health-related quality of life
ML: machine learning
NLP: natural language processing
PRISMA: Preferred Reporting Items for Systematic Reviews and Meta-Analyses
PRO: patient-reported outcome
RCT: randomized controlled trial
RoB 2: revised Cochrane risk of bias 2
ROBINS-I V2: Risk of Bias in Non-Randomized Studies—of Interventions, Version 2
VR: virtual reality
