# Supplementary material for: Patient-Facing AI-Enabled Digital Health Technologies and Quality of Life in Cancer: Systematic Review and Exploratory Meta-Analysis
Source: JMIR Mhealth Uhealth. 2026 Aug 10;14:e94793. doi: 10.2196/94793 (PMC13456306; doi:10.2196/94793)
Supplement: Supplementary file 2 [file mhealth-v14-e94793-s001.docx]

**Multimedia Appendix A**. HRQOL domain comparison.

| Study | Zhang, 2025 [1] | Jiang, 2024 [2] | Schmitz, 2023 [3] |
| --- | --- | --- | --- |
| Instrument | **EORTC QLQ-C30 [4]** | **FACT-B [5]** | **SF-36 [6]** |
| Cancer | **Mixed** | **Breast** | **Breast** |
| General health | Global health status / QoL (2 items) | FACT-G total score (27 items) | General health (5 items): perceptions and expectations about health |
| Physical functioning | Physical functioning (5 items): walking, self-care, daily activities, stairs, mobility | Physical well-being (7 items): energy, nausea, fatigue, pain, sleep, physical symptoms interference | Physical functioning (10 items): vigorous/moderate activities, lifting, walking, bathing/dressing |
| Role functioning | Role functioning (2 items): work/household tasks limitations | Functional well-being (7 items): work, daily activities, enjoyment, sleep, leisure, life satisfaction | Role limitations due to physical health (4 items): problems with work/daily activities due to physical health |
| Emotional well-being | Emotional functioning (4 items): tension, worry, irritability, depression | Emotional well-being (6 items): sadness, coping, hope, anxiety, worry | Emotional well-being (5 items): anxiety, depression, happiness, calmness, psychological well-being |
| Social functioning | Social functioning (2 items): interference with family/social activities | Social/Family well-being (7 items): family support, social interactions, relationships, support network | Social functioning (2 items): interference with social activities due to health |

Note: This table summarizes the HRQOL domains that were considered conceptually comparable across instruments. The number of items and domain descriptions were extracted from instrument manuals and supporting documentation to provide an overview of how domains were grouped for the current meta-analysis. Item counts and content reflect the standard structure of each instrument and may not correspond precisely to the versions or items used in the individual studies. Some instruments included additional domains that could not be compared across the included studies. These domains are omitted from this table.

**References**

1.	Zhang J, lv XN, Wang M, Zhang J, Qi F. Continuous nursing symptom management in cancer chemotherapy patients using deep learning. *Sci Rep*. 2025;15(1):7990. doi:10.1038/s41598-025-92762-7

2.	Jiang L, Xu J, Wu Y, Liu Y, Wang X, Hu Y. Effects of the “AI-TA” Mobile App With Intelligent Design on Psychological and Related Symptoms of Young Survivors of Breast Cancer: Randomized Controlled Trial. *JMIR Mhealth Uhealth*. 2024;12:e50783. doi:10.2196/50783

3.	Schmitz KH, Kanski B, Gordon B, et al. Technology-based supportive care for metastatic breast cancer patients. *Support Care Cancer*. 2023;31(7):401. doi:10.1007/s00520-023-07884-3

4.	Questionnaires. EORTC - Quality of Life. Accessed March 3, 2026. https://qol.eortc.org/questionnaires/

5.	FACT-B. FACIT Group. Accessed March 3, 2026. https://www.facit.org/measures/fact-b

6.	36-Item Short Form Survey (SF-36) Scoring Instructions. RAND. Accessed March 3, 2026. https://www.rand.org/health/surveys/mos/36-item-short-form/scoring.html
