# Supplementary material for: Patient-Facing AI-Enabled Digital Health Technologies and Quality of Life in Cancer: Systematic Review and Exploratory Meta-Analysis
Source: JMIR Mhealth Uhealth. 2026 Aug 10;14:e94793. doi: 10.2196/94793 (PMC13456306; doi:10.2196/94793)
Supplement: Supplementary file 3 [file mhealth-v14-e94793-s002.docx]

**Multimedia Appendix B**. Changes in patient-reported outcomes.

| **Outcome category** | **Study** | **Outcome measure** | **Effect size / Magnitude** | **Interpretation of change** |
| --- | --- | --- | --- | --- |
| Quality of life | Buchan, 2024 [1] | Rating of quality of life (1-7 scale, higher=better) | 75.3% rated 4-6; mean 4.9/7 |  |
|  | Buchan, 2024 [1] | Extent of improvement in quality of life (1-5 scale, higher=better) | 81.4% rated 3-5 |  |
|  | Horesh, 2022 [2] | Physical quality of life (WHOQOL-BREF) | Pre 11.93 (SD 2.0) → Post 15.36 (SD 2.47), d=-1.49, p<0.001 | Improvement |
|  | Horesh, 2022 [2] | Psychological quality of life (WHOQOL-BREF) | Pre 13.66 (SD 2.65) → Post 14.47 (SD 2.67) d=-0,30, p<0.01 | Improvement |
|  | Horesh, 2022 [2] | Environmental quality of life (WHOQOL-BREF) | No significant changes | No change |
|  | Horesh, 2022 [2] | Social quality of life (WHOQOL-BREF) | No significant changes | No change |
|  | Hu, 2025 [3] | Quality of life (FACT-B) | T0: int 92.00 (SE 2.26) vs ctrl 87.54 (SE 1.95), p=0.141; T1: int 105.44 (SE 1.41) vs ctrl 101.14 (SE 1.53), p=0.041; T2: int 120.82 (SE 1.39) vs ctrl 110.23 (SE 1.51), p<0.001; Significant time, group, and time × group interaction effect  (β = 15.384, 95% CI, 13.028–17.740; p<0.001) | Improvement |
|  | Jiang, 2024 [4] | Quality of life (FACT-B) | T0: int 90.02 (SD 12.44) vs ctrl 92.48 (SD 15.15), p=0.52; T1: int 106.68 (SD 12.49) vs ctrl 100.33 (SD 12.32), p=0.01; T2: int 124.47 (SD 9.14) vs ctrl 113.50 (SD 11.20), p<0.001; Significant group (p=0.02), time (p<0.001), and group × time interaction effect (p<0.001) | Significant differences between groups were found both in T1 and T2 |
|  | Jiang, 2024 [4] | Physical well-being | T0: int 20.04 (SD 5.05) vs ctrl 20.50 (SD 4.27), p=0.65; T1: int 21.61 (SD 4.44) vs ctrl 21.17 (SD 3.66), p=0.45; T2: int 24.91 (SD 3.00) vs ctrl 22.00 (SD 3.57), p<0.001 | Significant differences between groups were found only at T2 |
|  | Jiang, 2024 [4] | Social or family well-being | T0: int 20.11 (SD 7.93) vs ctrl 19.86 (SD 5.07), p=0.09; T1: int 21.31 (SD 4.37) vs ctrl 20.52 (SD 3.76), p=0.39; T2: int 24.89 (SD 2.88) vs ctrl 21.45 (SD 3.47), p<0.001 | Significant differences between groups were found only at T2 |
|  | Jiang, 2024 [4] | Emotional well-being | T0: int 12.87 (SD 4.28) vs ctrl 12.88 (SD 4.27), p=0.98; T1: int 15.98 (SD 2.57) vs ctrl 15.45 (SD 3.01), p=0.39; T2: int 18.50 (SD 2.57) vs ctrl 17.24 (SD 2.99), p<0.001 | Significant differences between groups were found only at T2 |
|  | Jiang, 2024 [4] | Functional well-being | T0: int 12.89 (SD 6.75) vs ctrl 12.57 (SD 5.11), p=0.81; T1: int 17.86 (SD 4.72) vs ctrl 15.55 (SD 3.92), p=0.008; T2: int 23.85 (SD 3.84) vs ctrl 18.29 (SD 3.63), p<0.001 | Significant differences between groups were found both in T1 and T2 |
|  | Jiang, 2024 [4] | Additional concerns | T0: int 26.91 (SD 5.85) vs ctrl 26.67 (SD 3.69), p=0.57; T1: int 29.92 (SD 4.78) vs ctrl 27.64 (SD 3.35), p=0.02; T2: int 32.31 (SD 3.29) vs ctrl 28.52 (SD 3.38), p<0.001 | Significant differences between groups were found both in T1 and T2 |
|  | Kamdar, 2024 [5] | Quality of life (FACT-G) | No significant differences between the two groups longitudinally | No difference |
|  | Li, 2025 [6] | Global health status (EORTC QLQ-C30) | Int +16.25 (SD 23.02) vs ctrl +1.04 (SD 13.90), p=0.03 | Improvement |
|  | Li, 2025 [6] | Physical functioning | Int +6.00 (SD 8.06) vs ctrl -1.67 (SD 15.3), p=0.11 | No difference |
|  | Li, 2025 [6] | Role functioning | Int +6.67 (SD 13.68) vs ctrl +9.38 (SD 21.05), p=0.58 | No difference |
|  | Li, 2025 [6] | Emotional functioning | Int +4.58 (SD 13.10) vs ctrl -0.52 (SD 16.52), p=0.24 | No difference |
|  | Li, 2025 [6] | Cognitive functioning | Int 0.00 (SD 10.81) vs ctrl -6.25 (SD 13.44), p=0.13 | No difference |
|  | Li, 2025 [6] | Social functioning | Int +11.67 (SD 18.81) vs ctrl +3.13 (SD 20.38), p=0,32 | No difference |
|  | Schmitz, 2023 [7] | General health (SF-36) | Int +0.76 (SE 11.98) vs ctrl -0.50 (SE 12.97), p=0.7567 | No change |
|  | Schmitz, 2023 [7] | Vitality/fatigue (SF-36) | Int +6.67 (SE 12.83) vs ctrl +2.00 (SE 17.35), p=0.3494 | No change |
|  | Schmitz, 2023 [7] | Physical functioning (SF-36) | Int +8.63 (SE 19.03) vs ctrl 0.00 (SE 10.39), p=0.1230 | No change |
|  | Schmitz, 2023 [7] | Role limitations due to physical health (SF-36) | Int +6.94 (SE 49.86) vs ctrl 0.00 (SE 35.36), p=0.6274 | No change |
|  | Schmitz, 2023 [7] | Role limitations due to emotional problems (SF-36) | Int +16.67 (SE 56.30) vs ctrl -1.67 (SE 41.48), p=0.2332 | No change |
|  | Schmitz, 2023 [7] | Emotional well-being (SF-36) | Int +0.44 (SE 12.72) vs ctrl +0.40 (SE 7.33), p=0.9897 | No change |
|  | Schmitz, 2023 [7] | Social functioning (SF-36) | Int +1.39 (SE 17.62) vs ctrl 1.25 (SE 13.99), p=0.9789 | No change |
|  | Schmitz, 2023 [7] | Pain (SF-36) | Int +3.61 (SE 19.18) vs ctrl -2.00 (SE 18.65), p=0.3677 | No change |
|  | Zhang, 2025 [8] | Overall [global] health status (EORTC QLQ-C30) | Int: Pre 53.05 (SD 10.3) → Post 68.3 (SD 10.6), p<0.01 Ctrl: Pre 52.5 (SD 11.4) → Post 56.8 (SD 10.9), p=0.785 | Between-group differences are poorly reported. However, the authors narratively report greater improvements in the intervention group compared with the control group (p<0.05). |
|  | Zhang, 2025 [8] | Somatic [physical] functioning (EORTC QLQ-C30) | Int: Pre 57.3 (SD 11.2) → Post 64.7 (SD 11.5), p<0.01 Ctrl: Pre 56.6 (SD 11.7) → Post 63.7 (SD 11.3), p=0.61 | Between-group differences are poorly reported. However, the authors narratively report no statistically significant differences between intervention and control groups (p>0.05). |
|  | Zhang, 2025 [8] | Role functioning (EORTC QLQ-C30) | Int: Pre 61.1 (SD 12.1) → Post 77.5 (SD 12.6), p<0.01 Ctrl: Pre 60.6 (SD 12.8) → Post 68.8 (SD13.2), p=0.71 | Between-group differences are poorly reported. However, the authors narratively report greater improvements in the intervention group compared with the control group (p<0.05). |
|  | Zhang, 2025 [8] | Cognitive functioning (EORTC QLQ-C30) | Int: Pre 60.2 (SD 12.7) → Post 81.1 (SD 13.1), p<0.01 Ctrl: Pre 59.6 (SD 11.6) → Post 71.3 (SD 12.4), p=0.65 | Between-group differences are poorly reported. However, the authors narratively report greater improvements in the intervention group compared with the control group (p<0.05). |
|  | Zhang, 2025 [8] | Emotional functioning (EORTC QLQ-C30) | Int: Pre 60.7 (SD 11.8) → Post 82.6 (SD 12.1), p<0.01 Ctrl: Pre 60.1 (SD 12.3) → Post 71.6 (SD 11.7), p=0.665 | Between-group differences are poorly reported. However, the authors narratively report greater improvements in the intervention group compared with the control group (p<0.05). |
|  | Zhang, 2025 [8] | Social functioning (EORTC QLQ-C30) | Int: Pre 49.9 (SD 11.3) → Post 71.3 (SD 10.9), p<0.01 Ctrl: Pre 50.6 (SD 10.9) → Post 59.4 (SD 11.2), p=0.045 | Between-group differences are poorly reported. However, the authors narratively report greater improvements in the intervention group compared with the control group (p<0.05). |
| Satisfaction and adherence | Buchan, 2024 [1] | Platform satisfaction (1-5 scale) | 93.6% rated 3-5; mean 4.0/5 |  |
|  | Buchan, 2024 [1] | Tips helpfulness (1-5 scale) | 98.3% rated 3-5 |  |
|  | Buchan, 2024 [1] | Recipes helpfulness (1-5 scale) | 97.3% rated 3-5 |  |
|  | Buchan, 2024 [1] | Platform accuracy (1-5 scale) | 93.7% rated 3-5 |  |
|  | Buchan, 2024 [1] | Tips utilization | 83.9% used tips |  |
|  | Buchan, 2024 [1] | Recipe utilization | 46.7% used recipes |  |
|  | Horesh, 2022 [2] | Perceived helpfulness | 70% found Frosty helpful |  |
|  | Horesh, 2022 [2] | Overall enjoyment | 97% enjoyed Frosty |  |
|  | Horesh, 2022 [2] | Daily use intention | 84% would use Frosty daily |  |
|  | Horesh, 2022 [2] | Recommendation likelihood | 97% would recommend Frosty |  |
|  | Hu, 2025 [3] | Adherence | 96.77% |  |
|  | Kamdar, 2024 [5] | Ease of understanding (0-10 scale) | Mean = 8.6 (SD 1.8) |  |
|  | Kamdar, 2024 [5] | Ease of use (0-10 scale) | Mean = 8.63 (SD 1.66) |  |
|  | Kamdar, 2024 [5] | Usefulness (0-10 scale) | Mean = 7.8 (SD 2.4) |  |
|  | Kamdar, 2024 [5] | Weekly pain reporting | 86% → 48% (week 8) | Decline |
|  | Li, 2025 [6] | Adherence rate | 88 % |  |
|  | Li, 2025 [6] | Session completion rate | 101.2% |  |
|  | Schmitz, 2023 [7] | Overall satisfaction | ≥70% satisfied with all interventions |  |
|  | Schmitz, 2023 [7] | Client Satisfaction Questionnaire (8-32 scale, higher=better) | Mean = 25.36 (SD 4.87) |  |
|  | Schmitz, 2023 [7] | Found logical (1-10 scale, higher=better) | Mean = 7.42 (SD 1.62) |  |
|  | Schmitz, 2023 [7] | Helped with symptoms (1-10 scale, higher=better) | Mean = 5.76 (SD 2.50) |  |
|  | Schmitz, 2023 [7] | Would recommend to friend (1-10 scale, higher=better) | Mean = 7.24 (SD 2.36) |  |
|  | Schmitz, 2023 [7] | Improvement in symptoms (0-100 scale, higher=better) | Mean = 47.81 (SD 28.82) |  |
|  | Schmitz, 2023 [7] | Usability (0-100 scale, higher=better) | Mean = 86.14 (SD 11.69) |  |
|  | Schmitz, 2023 [7] | Engagement (≥30/90 days login) | 64.9% |  |
|  | Schmitz, 2023 [7] | Use | 58% (month 4) → 47% (month 5) → 29% (month 6) | Decline |
|  | Zhang, 2025 [8] | Platform satisfaction (range not specified, see Table 4 for more information) | 4.93 ± 0.13 |  |
|  | Zhang, 2025 [8] | Ease of use and access | 85 % |  |
|  | Zhang, 2025 [8] | Technology challenges (accessibility of platform, internet, and content) | 5-15% reported difficulties |  |
| Mental health | Horesh, 2022 [2] | Perceived Stress Scale (PSS, 0-40 scale) | Pre 21.5 (SD 3.90) → Post 19.34 (SD 3.16), d=-0.61, p=0.005 | Improvement |
|  | Horesh, 2022 [2] | General Psychological Distress (K-10, 10-50 scale) | Pre 20.81 (SD 6.71) → Post 17.27 (SD 5.64), d=0.57, p<0.01 | Improvement |
|  | Horesh, 2022 [2] | Illness Perception (B-IPQ) | Pre 41.64 (SD 14.64) → Post 33.51 (SD 4.5), d=0.62, p<0.01 | Improvement |
|  | Hu, 2025 [3] | Psychological frailty (MSAS-SF, 0-4 scale) | T0: int 1.46 (SE 0.09) vs ctrl 1.58 (SE 0.07), p=0.328; T1: int 0.80 (SE 0.04) vs ctrl 1.17 (SE 0.06), p<0.001; T2: int 0.40 (SE 0.03) vs ctrl 0.95 (SE 0.05), p<0.001 Significant time, group, and time × group interaction effects (β =−0.394, 95% CI, −0.469 to −0.320; p<0.001) | Greater decrease (improvement) in the intervention group |
|  | Jiang, 2024 [4] | Psychological distress (MSAS-SF, 0-4 scale) | T0: int 1.56 (SD 0.47) vs ctrl 1.51 (SD 0.50), p=0.25; T1: int 1.17 (SD 0.46) vs ctrl 1.23 (SD 0.37), p<0.001; T2: int 0.68 (SD 0.21) vs ctrl 0.91 (SD 0.22), p<0.001 | Greater decrease (improvement) in the intervention group |
|  | Jiang, 2024 [4] | Psychological frequency (symptom frequency) | T0: int 1.43 (SD 0.36) vs ctrl 1.52 (SD 0.29), p=0.67;  T1: int 1.41 (SD 0.19) vs ctrl 1.45 (SD 0.14), p=0.006; T2: int 0.83 (SD 0.13) vs ctrl 0.95 (SD 0.21), p<0.001 | Greater decrease (improvement) in the intervention group |
|  | Jiang, 2024 [4] | Psychological distress (sub) | T0: int 1.48 (SD 0.11) vs ctrl 1.59 (SD 0.14), p=0.38; T1: int 0.79 (SD 0.25) vs ctrl 1.22 (SD 0.12), p<0.001; T2: int 0.40 (SD 0.16) vs ctrl 0.93 (SD 0.17), p<0.001 | Greater decrease (improvement) in the intervention group |
|  | Jiang, 2024 [4] | Global distress | T0: int 1.30 (SD 0.17) vs ctrl 1.46 (SD 0.26), p=0.07; T1: int 0.94 (SD 0.23) vs ctrl 1.23 (SD 0.11), p<0.001; T2: int 0.41 (SD 0.20) vs ctrl 1.03 (SD 0.13), p<0.001 | Greater decrease (improvement) in the intervention group |
|  | Kamdar, 2024 [5] | Anxiety (GAD-7, 0-21, higher=worse) | Baseline: int 6.67 (SD 5.88) vs ctrl 5.9 (SD 4.85), p=0.700;  4 weeks: int 6.71 (SD 5.07) vs ctrl 5.03 (SD 3.67), p=0.260;  8 weeks: int 7.68 (SD 4.96) vs ctrl 5.03 (SD 3.6), p=0.022; Longitudinal model, between group: β=0.21 (95% CI 0.039, 0.37, p=0.015) | Worsening |
|  | Li, 2025 [6] | Anxiety (Hospital Anxiety and Depression Scale) | Int -0.31 (SD 0.44) vs ctrl -0.05 (SD 0.29), p=0.048 | Improvement |
|  | Schmitz, 2023 [7] | Distress (practical, family, emotional, spiritual, physical) | No significant between-group differences | No change |
|  | Zhang, 2025 [8] | Anxiety (SAS, higher=worse) | Int: Pre 56.4 (SD 1.8) → Post 41.3 (SD 2.1), p<0.01 Ctrl: Pre 55.3 (SD 1.3) → Post 51.6 (SD 2.4), p<0.01 | Between-group differences are poorly reported. However, the authors narratively report greater improvements in the control group compared with the intervention group (p<0.05). |
|  | Zhang, 2025 [8] | Depression (SDS, higher=worse) | Int: Pre 60.1 (SD 2.3) → Post 34.2 (SD 2.6), p<0.01 Ctrl: Pre 59.7 (SD 2.5) → Post 51.4 (SD 2.7), p<0.01 | Between-group differences are poorly reported. However, the authors narratively report greater improvements in the intervention group compared with the control group (p<0.05). |
| Physical symptoms | Buchan, 2024 [1] | Symptom management help (1-5 scale) | 87.7% rated 3-5 |  |
|  | Buchan, 2024 [1] | Symptom reduction (≥2 surveys) | 32.4% reduction in symptom count;  34.0% reduction in total symptom score |  |
|  | Horesh, 2022 [2] | Hot Flash Interference (HFRDIS, 10-100 scale) | Pre 59.51 (SD 28.27) → Post 48.05 (SD 26.18), d=0.42, p<0.01 | Improvement |
|  | Horesh, 2022 [2] | Hot Flash Frequency (Hot Flash Rating Scale) | Pre 11.31 (SD 8.54) → Post 6.83 (SD 4.94), d=0.60, p<0.01 | Improvement |
|  | Horesh, 2022 [2] | Sleep Quality (total PSQI, 0-21, higher = worse) | Pre 8.32 (SD 3.92) → Post 6.31 (SD 3.74), d=0.52, p<0.01 | Improvement |
|  | Horesh, 2022 [2] | Sleep onset latency (PSQI component, higher = worse) | Pre 1.08 (SD 0.953) → Post 0.675 (SD 0.78), d=0.46, p<0.01 | Improvement |
|  | Horesh, 2022 [2] | Subjective sleep quality (PSQI component, higher = worse) | Pre 1.07 (SD 0.72) → Post 0.50 (SD 0.74), d=0.81, p>0.01 [possible typo] | Improvement |
|  | Hu, 2025 [3] | Fatigue (BFI, 0-10 scale) | T0: int 2.87 (SE 0.26) vs ctrl 3.43 (SE 0.18), p=0.088; T1: int 2.28 (SE 0.18) vs ctrl 3.14 (SE 0.11), p<0.001;  T2: int 1.88 (SE 0.10) vs ctrl 2.40 (SE 0.08), p<0.001; Significant group and time effects; ITT showed greater fatigue reduction vs control (β = 0.685, 95% CI 0.448–0.922; p<0.001) | Improvement |
|  | Hu, 2025 [3] | Physical activity (IPAQ-SF, MET scores) | T0: int 1042.55 (SE 287.77) vs ctrl 876.94 (SE 127.64), p=0.611; T1: int 1194.14 (SE 139.41) vs ctrl 888.95 (SE 124.84), p=0.108; T2: int 1294.40 (SE 170.65) vs ctrl 842.17 (SE 109.66), p=0.031; Significant group effect only (β = −378.71, 95% CI −705.24 to −52.18; p=0.023) | Improvement |
|  | Hu, 2025 [3] | Physical symptoms (MSAS-SF) | T0: int 0.86 (SE 0.06) vs ctrl 1.08 (SE 0.07), p=0.024; T1: int 0.58 (SE 0.05) vs ctrl 0.79 (SE 0.05), p=0.002; T2: int 0.33 (SE 0.04) vs ctrl 0.53 (SE 0.04), p<0.001; Significant group and time effects favoring ITT (β = 0.207, 95% CI 0.105–0.310; P < 0.001) | Improvement |
|  | Jiang, 2024 [4] | Physical distress (symptom burden) | T0: int 1.16 (SD 0.10) vs ctrl 1.08 (SD 0.15), p=0.30; T1: int 0.56 (SD 0.25) vs ctrl 0.75 (SD 0.14), p=0.002; T2: int 0.32 (SD 0.12) vs ctrl 0.49 (SD 0.15), p<0.001 | Improvement |
|  | Kamdar, 2024 [5] | Pain severity (BPI, 0-10, higher=worse) | Baseline: int 3.74 (SD 1.88) vs ctrl 4.02 (SD 1.67), p=0.41; 4 weeks: int 3.16 (SD 1.78) vs ctrl 4.28 (SD 1.99), p=0.010;  8 weeks: int 2.99 (SD 2.06) vs ctrl 4.05 (SD 1.78), p=0.017; Longitudinal model, between group: β=-0.09 (95% CI -0.17 to -0.007; p=0.034) | Improvement |
|  | Kamdar, 2024 [5] | Pain management barriers (BQ-II) | Baseline: int 1.30 (SD 0.82) vs ctrl 1.18 (SD 0.82), p=0.430; 4 weeks: int 0.96 (SD 0.85) vs ctrl 1.00 (SD 0.82), p=0.790; 8 weeks: int 1.00 (SD 0.84) vs ctrl 1.15 (SD 0.89), p=0.480; Longitudinal model, between group: β=–0.037 (95& CI –0.072 to –0.001; p=0.042) | Improvement |
|  | Kamdar, 2024 [5] | Clinically significant pain improvement | Int 35.7% vs ctrl 16.3%, p=0.041 | Higher improvement in the intervention group |
|  | Li, 2025 [6] | Fatigue (Multidimensional Fatigue Symptom Inventory—Short Form) | Int -8.89 (SD 15.96) vs ctrl +1.39 (SD 12.09), p=0.02 | Improvement |
|  | Li, 2025 [6] | Daytime dysfunction (PSQI) | Int -0.55 (SD 0.69) vs ctrl 0.00 (0.52), p=0.02 | Improvement |
|  | Schmitz, 2023 [7] | Vitality/fatigue, pain, sleep | No significant between-group differences | No change |
| Other PROs | Jiang, 2024 [4] | Self-Efficacy (CBI-B, 12-108) | T0: int 36.13 (SD 6.72) vs ctrl 37.07 (SD 3.50), p=0.08; T1: int 82.83 (SD 5.70) vs ctrl 65.35 (SD 3.06), p<0.001; T2: int 92.83 (SD 3.04) vs ctrl 85.65 (SD 2.79), p<0.001 | Improvement |
|  | Hu, 2025 [3] | Social Support (SSRS, higher=better) | T0: int 36.29 (SE 1.04) vs ctrl 39.59 (SE 0.97), p=0.022; T1: int 42.43 (SE 1.00) vs ctrl 43.14 (SE 0.85), p=0.593; T2: int 49.57 (SE 0.67) vs ctrl 46.59 (SE 0.83), p=0.006; Significant time and time × group interaction effects (β = 7.145, 95% CI, 6.053–8.237; p<0.001) | Improvement |
|  | Jiang, 2024 [4] | Social Support (SSRS, 22-66, higher=better) | T0: int 38.04 (SD 8.19) vs ctrl 39.33 (SD 39.33), p=0.07; T1: int 42.67 (SD 7.94) vs ctrl 42.31 (SD 6.68), p=0.73; T2: int 50.09 (SD 4.95) vs ctrl 45.10 (SD 6.44), p=0.002 | Improvement |

Notes: Data are reported as presented in the original publications. Some studies report pre- and post-intervention values, whereas others report only the change in scores.

Abbreviations: Ctrl, control group; Int, intervention group; PROs, patient-reported outcomes; SD, standard deviation; SE, standard error.

**References**

1.	Buchan ML, Goel K, Schneider CK, Steullet V, Bratton S, Basch E. National Implementation of an Artificial Intelligence–Based Virtual Dietitian for Patients With Cancer. *JCO Clin Cancer Inform*. 2024;(8):e2400085. doi:10.1200/CCI.24.00085

2.	Horesh D, Kohavi S, Shilony-Nalaboff L, et al. Virtual Reality Combined with Artificial Intelligence (VR-AI) Reduces Hot Flashes and Improves Psychological Well-Being in Women with Breast and Ovarian Cancer: A Pilot Study. *Healthcare (Basel)*. 2022;10(11). doi:10.3390/healthcare10112261

3.	Hu Y, Wiley J, Jiang L, et al. Digital humanistic program to manage premature frailty in young breast cancer survivors with gender perspective. *NPJ digital medicine*. 2025;8(1):35. doi:10.1038/s41746-025-01439-y

4.	Jiang L, Xu J, Wu Y, Liu Y, Wang X, Hu Y. Effects of the “AI-TA” Mobile App With Intelligent Design on Psychological and Related Symptoms of Young Survivors of Breast Cancer: Randomized Controlled Trial. *JMIR Mhealth Uhealth*. 2024;12:e50783. doi:10.2196/50783

5.	Kamdar M, Jethwani K, Centi A, et al. A digital therapeutic application (ePAL) to manage pain in patients with advanced cancer: a randomized controlled trial. *Journal of pain and symptom management*. 2024;68(3):261‐271. doi:10.1016/j.jpainsymman.2024.05.033

6.	Li G, Zhou X, Deng J, et al. Digital Therapeutics–Based Cardio-Oncology Rehabilitation for Lung Cancer Survivors: Randomized Controlled Trial. *JMIR Mhealth Uhealth*. 2025;13:e60115. doi:10.2196/60115

7.	Schmitz KH, Kanski B, Gordon B, et al. Technology-based supportive care for metastatic breast cancer patients. *Support Care Cancer*. 2023;31(7):401. doi:10.1007/s00520-023-07884-3

8.	Zhang J, lv XN, Wang M, Zhang J, Qi F. Continuous nursing symptom management in cancer chemotherapy patients using deep learning. *Sci Rep*. 2025;15(1):7990. doi:10.1038/s41598-025-92762-7
