# Supplementary material for: Patient-Facing AI-Enabled Digital Health Technologies and Quality of Life in Cancer: Systematic Review and Exploratory Meta-Analysis
Source: JMIR Mhealth Uhealth. 2026 Aug 10;14:e94793. doi: 10.2196/94793 (PMC13456306; doi:10.2196/94793)
Supplement: Supplementary file 4 [file mhealth-v14-e94793-s003.docx]

**Multimedia Appendix C**. Leave-one-out sensitivity analysis.

| **Study** | **Hedges’s g** | **95% CI** | **p-value** | |
| --- | --- | --- | --- | --- |
| **General health** | | | |  |
| Zhang, 2025 [1] | 0.581 | -0.446 to 1.609 | 0.267 | |
| Jiang, 2024 [2] | 0.582 | -0.438 to 1.602 | 0.264 | |
| Schmitz, 2023 [3] | 1.067 | 0.808 to 1.326 | 0.000 | |
| **Physical functioning** | | | |  |
| Zhang, 2025 [1] | 0.548 | -0.197 to 1.292 | 0.149 | |
| Jiang, 2024 [2] | 0.092 | -0.200 to 0.385 | 0.536 | |
| Schmitz, 2023 [3] | 0.477 | -0.300 to 1.254 | 0.229 | |
| **Role functioning** | | | |  |
| Zhang, 2025 [1] | 0.748 | -0.724 to 2.221 | 0.319 | |
| Jiang, 2024 [2] | 0.384 | -0.289 to 1.057 | 0.264 | |
| Schmitz, 2023 [3] | 1.064 | 0.275 to 1.854 | 0.008 | |
| **Emotional well-being** | | | |  |
| Zhang, 2025 [1] | 0.268 | -0.217 to 0.754 | 0.279 | |
| Jiang, 2024 [2] | 0.468 | -0.468 to 1.433 | 0.341 | |
| Schmitz, 2023 [3] | 0.690 | 0.231 to 1.149 | 0.003 | |
| **Social functioning** | | | |  |
| Zhang, 2025 [1] | 0.555 | -0.523 to 1.632 | 0.313 | |
| Jiang, 2024 [2] | 0.556 | -0.516 to 1.628 | 0.309 | |
| Schmitz, 2023 [3] | 1.073 | 0.814 to 1.333 | 0.000 | |

**References**

1.	Zhang J, lv XN, Wang M, Zhang J, Qi F. Continuous nursing symptom management in cancer chemotherapy patients using deep learning. *Sci Rep*. 2025;15(1):7990. doi:10.1038/s41598-025-92762-7

2.	Jiang L, Xu J, Wu Y, Liu Y, Wang X, Hu Y. Effects of the “AI-TA” Mobile App With Intelligent Design on Psychological and Related Symptoms of Young Survivors of Breast Cancer: Randomized Controlled Trial. *JMIR Mhealth Uhealth*. 2024;12:e50783. doi:10.2196/50783

3.	Schmitz KH, Kanski B, Gordon B, et al. Technology-based supportive care for metastatic breast cancer patients. *Support Care Cancer*. 2023;31(7):401. doi:10.1007/s00520-023-07884-3
